# Correction: Catalytic wet peroxide degradation of acrylonitrile wastewater by ordered mesoporous Ag/CeO_2_: synthesis, performance and kinetics

**DOI:** 10.1039/d4ra90006e

**Published:** 2024-01-24

**Authors:** Guozheng Zhao, Hui Liang, Hongzhu Xu, Changbo Li, Qingwei Tan, Daihang Zhang

**Affiliations:** a School of Environmental & Safety Engineering, Liaoning Petrochemical University Liaoning Fushun 113001 China lnpulcb@126.com

## Abstract

Correction for ‘Catalytic wet peroxide degradation of acrylonitrile wastewater by ordered mesoporous Ag/CeO_2_: synthesis, performance and kinetics’ by Guozheng Zhao *et al.*, *RSC Adv.*, 2021, **11**, 15959–15968. DOI: https://doi.org/10.1039/D1RA01258D.

The authors regret the omission of a funding acknowledgement in the original article. This acknowledgement is given below.

The project was supported by the China National Key Project of Science and Technology “Water Pollution Control and Governance” (2012ZX07202-002), Science and Technology research project of Liaoning Provincial Department of Education (L2020020), and the Talent Scientific Research Fund of LNPU (No. 2020XJJL-015).

The Royal Society of Chemistry apologises for these errors and any consequent inconvenience to authors and readers.

## Supplementary Material

